# Recurrent desmoid tumor of the abdominal wall

**Published:** 2012-11-20

**Authors:** Imane Toughrai, Aya Oufkir, Said Ait Laalim, Karim Ibn Majdoub, Khalid Mazaz

**Affiliations:** 1 Department B of general surgery, Hassan II faculty hospital, Fes, Morocco; 2 Department of plastic and reconstructive surgery, Hassan II faculty hospital, Fes, Morroco

**Keywords:** Desmoid tumor, recurrent tumor, abdominal wall, reconstruction

## Abstract

Desmoid tumors most often occur in abdominal wall. Their tendency to recur lead to repeated operations which can make the abdominal wall reconstruction difficult. We report a 28-year-old female history. The patient was referred to our hospital for a recurrent desmoid tumor of the abdominal wall. The tumor was totally removed. The reconstruction was successful and the patient recovery was uneventful. Radical surgery still the mainstay of the desmoid tumors treatement. In abdominal wall location, the reconstruction can be a real challenge. Many procedures are discussed in literature. We used a double layer mesh covered by a fascial bepedicled flap. Taking into account their unpredictable behaviour and treatment complications, recurrent abdominal desmoid tumors can be managed simply and efficiently.

## Introduction

Desmoid tumors constitute an aggressive form of fibromatosis, originating from muscle and aponeurosis. They account less than 3% of all soft tissue neoplasms [1, 2]. Abdominal wall is one of the main location of desmoid tumors. They don't metastasize, but they have tendency to recur. Repetitive surgery makes abdominal wall reconstruction difficult.

## Patient and observation

**Figure 1:**
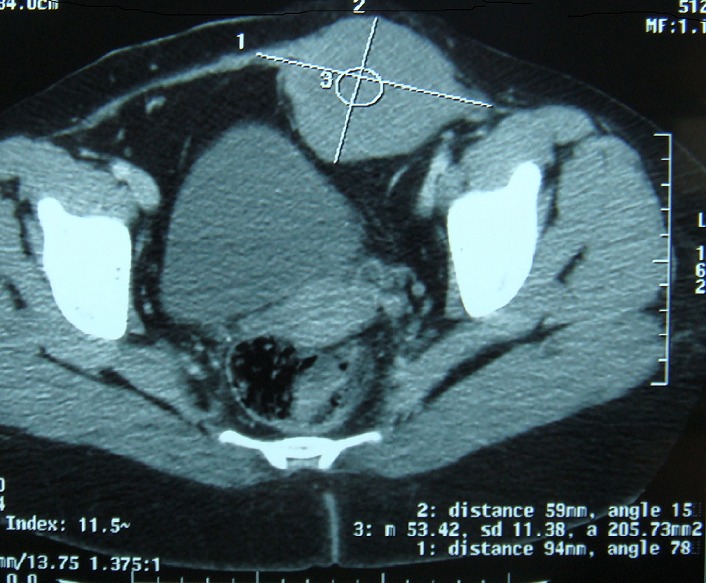
Abdominal CT scan shows an heterogeneous mass of the left iliaca fossa

**Figure 2:**
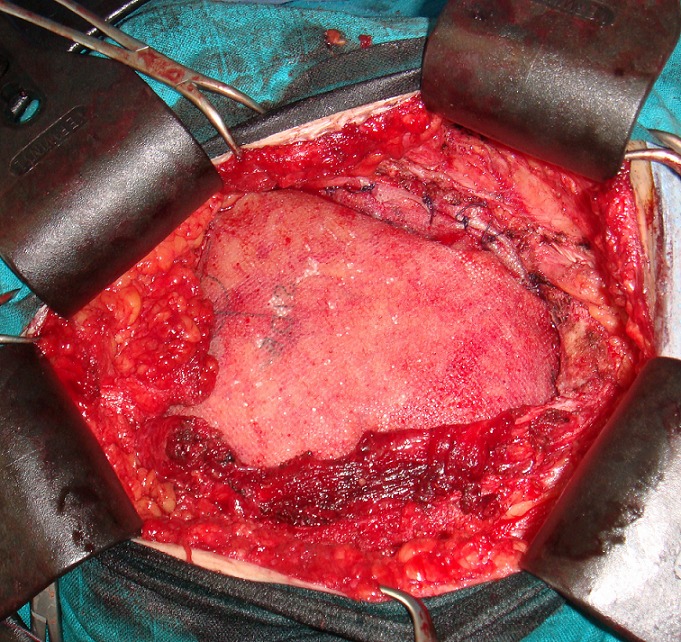
Operating view shows the abdominal wall defect reconstructed with a double layer mash

**Figure 3:**
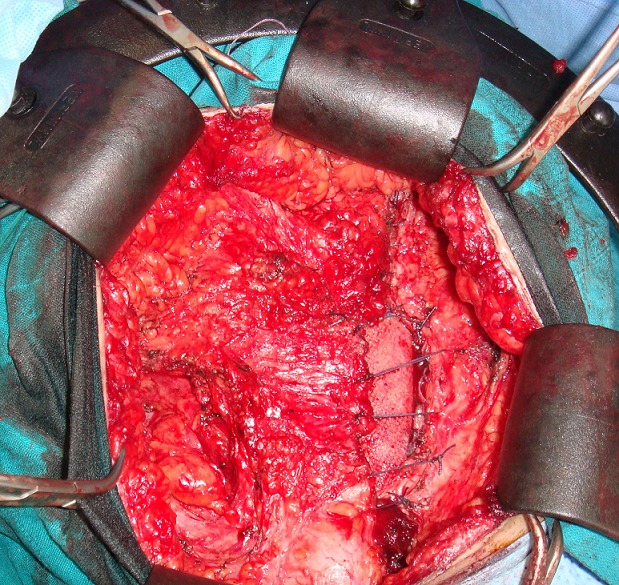
Operating view shows the coverage by a bepedicled fascial flap after reconstruction

A 28- year-old female who had a history of tow caesareans. She was, also, operated 8 months before for a desmoid tumor of the abdominal wall. She was referred to our hospital for a growing mass of the lower abdominal wall. Clinical examination revealed a 10 cm mass of the left iliaca fossa, which was fixed to the pubic bone. Abdominal computed tomography scan (CT scan) demonstrated an heterogeneous para-ombilical tumor involving the left rectus abdominis muscle (Figure 1). We concluded to a recurrent desmoid tumor of the abdominal wall. The surgery was performed within the existent midline abdominal incision. The mass was originating from the left rectus abdominis muscle, arising from the ombilicus to the pubic bone in which it was strongly adhered. It extended to the limit with the inguinal ligament, to abdominal transversal and oblique muscles and to bladder on its anterior surface. The tumor was totally removed respecting at least 2 cm of margin with healthy tissue. The resection included left rectus muscle and both oblique muscles. The resulting defect in the abdominal wall measured 15/15 cm. it was repaired by a double layer mesh which was covered by a bepedicled flap using the anterior fascia of the controlateral rectus abdominis, combined with an advancement of the posterior fascia after a dissection between the residual external and internal oblique muscles (Figure 2, Figure 3). The patient recovery was uneventful and she was discharged at the 5th postoperative day. Histological examination of the specimen showed a desmoid tumor with free margins. The outcome 13 months later didn't show any local recurrence or incisional hernia.

## Discussion

Abdominal desmoid tumor occur in the abdominal wall in 40 to 50% of cases [2, 3]. Especially in young women, with a history of pregnancy or previous surgery [1]. It was the case in our patient. The tumor development appeared to be associated with her second caesarean which preceded symptoms four months ago. Recurrence diagnosis of desmoid tumor in our patient was facilitated by her surgical history, tumor location within the anterior abdominal wall and by the CT scan findings. In other circumstances, definitive diagnosis must be established with histo-pathologic analysis [3, 4]. Desmoid tumor appearances in CT and MR imaging vary depending on their composition which changes over time [1, 2, 4].

Although they don't metastasize, their infiltrative growth and tendency to recur even after macroscopic complete excision cause real management challenge in desmoid tumors.

Surgery must be radical with the intent of achieving wide margins. This may leave extensive parietal loss of substance. However, it may not prevent recurrence which can occur in 20 to 30% of cases after complete resection. Recurrence rate can be more than 50% in resection R1 and R2 [5]. The median time to recurrence reported in literature is 14 to 17 months [5]. This situation leads to repeated operations. The quality of surgical excision is a major prognosis factor that influences tumor control and functional outcome, but not survival [5]. According to some authors, tumor stabilisation or regression after active phase of about 3 years is possible [1, 5]. In this regard, the aggressiveness of treatment must be taken into account and compared to the benignity of the disease.

Whether in, primary or recurrent desmoid tumor, the integrity of the abdominal wall must be restored after resection. Anatomical reconstruction by direct suture with release incision is possible [3]. Most often, prosthetic material is necessary to cover the defect. Newly developed material offers a tension free and secured repair. In our patient, we used a double layer mesh, a biomaterial with an anti-adhesive and inner surface acceptable for intra-peritoneal placement. We combined a Gibson and Micheau's procedures which are respectively an advancement of the fascia of rectus abdominis and oblique muscles [6, 7]. It's a simple and easy technique of coverage using a well vascularized tissue that can be associated with prosthetic repair. In greater loss of substance, it's recommended to use distant or free muscle flap.

The effectiveness of radiation and non surgical methods such as chemotherapy, hormonotherapy and anti-inflammatory treatment is not proven yet. These therapies are recommended in patient in whom resection is technically impossible or when repetitive surgery is necessary [4, 5].

Considering the unpredictable behaviour of desmoid tumor and treatment complications, some authors advocate a wait-and-see policy as an available therapeutic option in this disease [5].

## Conclusion

Surgery when it's possible is still the treatment of choice, whether in primary or recurrent abdominal wall desmoid tumor. The quality of resection is a main prognosis factor. Radical surgery results in significant loss of substance in abdominal wall which can be managed using many reconstruction techniques. Repair by a double layer mesh covered by a pedicled fascial flap seems to be simple and efficient method.
